# There Is More than One Way to Crack an Oyster: Identifying Variation in Burmese Long-Tailed Macaque (*Macaca fascicularis aurea*) Stone-Tool Use

**DOI:** 10.1371/journal.pone.0124733

**Published:** 2015-05-13

**Authors:** Amanda Tan, Say Hoon Tan, Dhaval Vyas, Suchinda Malaivijitnond, Michael D. Gumert

**Affiliations:** 1 Division of Psychology, School of Humanities and Social Sciences, Nanyang Technological University, Singapore, 637322, Singapore; 2 Graduate Degree Program in Ecology, Colorado State University, Fort Collins, Colorado, 80523, United States of America; 3 National Primate Research Centre of Thailand and Department of Biology, Faculty of Science, Chulalongkorn University, Bangkok, 10330, Thailand; University of Oxford, UNITED KINGDOM

## Abstract

We explored variation in patterns of percussive stone-tool use on coastal foods by Burmese long-tailed macaques (*Macaca fascicularis aurea*) from two islands in Laem Son National Park, Ranong, Thailand. We catalogued variation into three hammering classes and 17 action patterns, after examining 638 tool-use bouts across 90 individuals. Hammering class was based on the stone surface used for striking food, being face, point, and edge hammering. Action patterns were discriminated by tool material, hand use, posture, and striking motion. Hammering class was analyzed for associations with material and behavioural elements of tool use. Action patterns were not, owing to insufficient instances of most patterns. We collected 3077 scan samples from 109 macaques on Piak Nam Yai Island’s coasts, to determine the proportion of individuals using each hammering class and action pattern. Point hammering was significantly more associated with sessile foods, smaller tools, faster striking rates, smoother recoil, unimanual use, and more varied striking direction, than were face and edge hammering, while both point and edge hammering were significantly more associated with precision gripping than face hammering. Edge hammering also showed distinct differences depending on whether such hammering was applied to sessile or unattached foods, resembling point hammering for sessile foods and face hammering for unattached foods. Point hammering and sessile edge hammering compared to prior descriptions of axe hammering, while face and unattached edge hammering compared to pound hammering. Analysis of scans showed that 80% of individuals used tools, each employing one to four different action patterns. The most common patterns were unimanual point hammering (58%), symmetrical-bimanual face hammering (47%) and unimanual face hammering (37%). Unimanual edge hammering was relatively frequent (13%), compared to the other thirteen rare action patterns (<5%). We compare our study to other stone-using primates, and discuss implications for further research.

## Introduction

Tool use has been reported in a wide variety of animal species. From these reports, we know many animals use tools for a variety of functions, and employ tools of differing material characteristics and with different modes of actions [1, 2]. One form of tool use that has received more scientific attention recently, is the use of percussive stone tools on encased foods by non-human primates: West African chimpanzees (*Pan troglodytes verus*) [3–7]; robust capuchins *Sapajus libidinosus* [8, 9] and *S*. *xanthosternos* [10]; and Burmese long-tailed macaques (*Macaca fascicularis aurea*) [11, 12]. These findings have renewed speculation on the relevance of stone tools to understanding the origins of technology and culture [13–15].

There has been extensive study of variation in the material elements of stone-tool use, that are, variations in the types of foods processed with tools, and the tools and anvils used to process them. West African chimpanzees crack oil-palm nuts (*Elaeis guineenis*), panda nuts (*Panda oleosa*), and coula nuts (*Coula edulis*), and around Bossou, Guinea, it has been found that neighbouring communities may crack different species of nuts even though all species might be present in either community [16]. Oil-palm nuts are easiest to crack, followed by coula nuts, and panda nuts are the toughest to crack [3]. Related to nut hardness, chimpanzees select different sizes and materials of stones, and different types of anvils to process different species of nuts [3, 17, 18]. Chimpanzees in Bossou use smaller stones that were either granite, quartz, or diorite, to process soft oil-palm nuts, while the chimpanzees of neighbouring Diecké use larger stones, that were almost exclusively granite, on harder panda nuts [17, 19]. Anvils in Bossou also mostly comprised of loose rocks, while in Diecké, embedded rocks were used as anvils [17]. In the Taï forest, where both stone and wood hammers and anvils were used, chimpanzees selected heavier stones for harder panda nuts, and more likely cracked them on rock anvils than softer tree-root anvils [3]. In addition, as coula nuts became less hard as the season progressed, some groups of chimpanzees then used wooden club hammers more than stone hammers, as the former were more readily available [18].

Similar research has also been done on stone-tool-using populations of capuchin monkeys. Capuchins at Fazenda Boa Vista most commonly process *Astrocarpum campestre*, *Attala barreirensis*, *Orbignya* sp. and *Attalea* sp. palm nuts [20], while those at Fazenda Serra Grande also processed *Attalea*, and in addition processed *Syagrus* palm nuts, the legumes of *Hymenaea* plants, and *Cnidoscolus* nuts [10]. These food items differed in weight, shell thickness, and structural complexity, which resulted in differences in the nuts’ resistance to cracking [20]. Hammer stones used to crack these nuts varied in weight but averaged above 1kg [10, 21], and also differed in shape, and type of rock, most commonly sandstone or quartzite, which also differed in density [21]. At yet another capuchin site, Rio Grande do Norte, *Manihot* nuts that were not cracked at other sites were also found, and stones used to crack these nuts were found to be up to 14 times lighter than other hammer stones [22].

In contrast to the detail at which variation in material elements of stone-tool use has been studied, there are comparatively few or no detailed catalogs on behavioural actions employed during stone hammering. One example where researchers have identified variation in tool use actions was investigations of grip postures used by wild chimpanzees during nut pounding with stones or wood. Early research reported two types of stone hammering in chimpanzees [23]. Chimpanzees at Bossou handled small stones with a “cup grip”, while those in the Ivory Coast used a “pestle grip” with one or both hands to manipulate large stone and wooden hammers. This variation was later expanded, at Ivory Coast, to six different types of power grips that related to the size or material of the tool [24]. In capuchins, some individual variation in the kinematics of striking actions during nut cracking has been identified, such as differences in lifting movements, jumping during lifting, and the posturing of the tail during striking [25]. The sample size for this study was small (n = 4), but these findings indicate that capuchins could use several variants of action patterns to crack nuts that have yet to be more broadly explored.

There are a few examples of detailed study on the elements of behavioural actions within a single tool-use type. In another form of tool use, the ant-dipping behaviour of chimpanzees across 14 study sites, 2 techniques involving different behavioural actions have been identified [26–28]. These are the “pull-through” technique, where chimpanzees dip for ants with one hand, then use their other hand to collect the ants from the tools; and the “direct-mouthing” technique, where chimpanzees use their mouths to directly sweep or nibble ants from tools. The use of these techniques differs across sites [28], and at some sites, is related to the aggressiveness of ant species [26]. These studies illustrate the importance of recognizing variation in behavioural actions, as it can help us understand the roles of ecological and cultural factors on the formation of tool traditions.

The most notable example of a catalog of behavioural actions however, comes from 30-year longitudinal studies of stone handling in Japanese macaques (*Macaca fuscata*), a behaviour related to stone-tool use. Researchers have identified 45 different action patterns in how these animals were handling stones during non-functional play [29]. These detailed discriminations of behavioural patterns helped these researchers to uncover presumed cultural differences in the types of actions observed between several populations [29], variation across individuals [30], ecological influences on learning [31], and developmental aspects of acquiring the behaviour [32, 33].

In Burmese long-tailed macaques, work has been done on material variation, which is comparative to some of the work in chimpanzees and capuchins. Variation in the kinds of foods that are processed has been studied, and 47 different food species have been reported [34]. Despite this being the longest list of stone-processed food items by any stone-using primate, 83.2% of the food material found on the shore is from 4 genera, rock oysters (*Saccostrea cucullata*), nerite snails (*Nerita spp*.), drills (*Thais bitubercularis*), and tooth-lipped snails (*Monodonto labio*). The small amount of remaining food material is from numerous other bivalves, marine gastropods, crustaceans, and shore plant seeds (e.g., sea almonds, *Terminalia catappa*). This skewed pattern of shell collection towards a heavy use of a few species, while using a range of many, resembles the coastal foraging selection of many traditional human societies [35–38], which is based on foraging for the most easy to acquire types of foods of sufficient size to be nourishing [34]. Coastal shellfish differ in whether they are sessile or unattached, and sessile macaque foods are almost exclusively rock oysters. Unattached foods include motile bivalves, gastropods and crustaceans, as well as coastal plant seeds. Foods also differ in size, shape, thickness and toughness of shells, as well as their location in the littoral zone, and thus there are many factors that could influence how each food type is collected and processed.

From variation in material elements and behavioural actions of macaque tool use, it has been inferred that there are two basic hammering forms used by macaques, *axe hammering* and *pound hammering*. Tool form is identifiable by use-wear, and strongly relates to the kind of food being processed. Axe hammers were stone tools that were primarily used for sessile oysters and thus were found in rock oyster beds and most often showed use-wear on their points. Pound hammers were stone tools that were used for unattached foods and thus were found among the debris on anvils and showed use-wear most commonly on the stone’s face [12; 39]. Axe and pound hammering also appeared to differ across several material and behavioural elements [12]. Pound hammers were found to be significantly larger, heavier, and harder than axe hammers. Furthermore, when pound hammering, macaques typically handled stones with power grips (i.e. squeezed between the fingers and the palm) and with one or two hands, while tools for axing sessile oysters were held with precision grips (i.e., pinched between the fingers and thumb) and with one hand. Lastly, when axe-hammering, macaques struck targets at a faster rate and directed striking towards a wider range of spatial orientations relative to body position, than when pound hammering.

Axe and pound hammering are not always fully discriminable, because there is variation within these two forms of tools use, that can be ambiguous as to whether they can be defined as axe hammering or pound hammering. Although the dichotomy works well and covers the majority of variation in macaque tool use in a broad sense, we can still explore in more detail the patterns of macaque tool-use variation. For example, other variations have been noted, that involve variation in hand use, postures, and striking actions [12]. However, detailed investigation of these variations has not yet been done. Also there are clearly different kinds of tool use, such as the use of shells as tools and the use of stones like fulcrums, which involves lifting only one end of the tool to strike food targets, while the other side remains on the anvil surface [12]. These, and other, tool use action patterns need to be better explored and documented to build a complete catalog of macaque tool-use actions.

In this paper, we present a tested classification system of both material and behavioural variation. Here, we have created a system to categorize variation in macaque tool use, and defined a set of hammering classes and tool-use action patterns employed by the Burmese long-tailed macaques of Laem Son National Park, Thailand. After identifying this behavioural variation, we then tested for associations between material and behavioural elements. We then used our new system to assess the usefulness of the previously described axe/pound hammering categorization of long-tailed macaque stone tools [12]. Finally, we examined the prevalence of each type of variation using behavioural observations. Overall, our goal was to establish a research tool for stronger tests of how learning processes, ecological differences, and geographical distribution affect macaque tool use, allowing better testing of hypotheses on the evolution and development of traditions in animals than have been possible in other primate models. As new populations of stone-tool-using Burmese long-tailed macaques are continuing to be discovered across Thailand and Myanmar [Gumert et al., in prep], and research on development is underway [Tan et al., in prep], a well-developed classification system of macaque tool-use actions, such as ours, will allow researchers to more clearly document patterns of variation and change.

## Methods

### Research and Ethical Permissions

We obtained appropriate permissions for our research. Our research was approved by, and in accordance with the ethical guidelines of the NTU-Institutional Animal Care & Use Committee in Singapore (ref: ARF SBS/NIE-A 0138 NZ; ARF SBS/NIE-A 0058; ARF SBS/NIE 0138 AZ). Furthermore, the National Research Council of Thailand provided permission to conduct research in Thailand (Project 2314), and the Department of National Parks, Wildlife, and Plant Conservation permitted research in Laem Son National Park.

### Study Site

We observed tool-using behaviour and collected video of tool use from two adjacent islands in Laem Son National Park, Ranong Province, Thailand, Piak Nam Yai (PNY) Island (9°34’48”N, 98°28’00’E) and Thao Island (9°34’41”N, 98°28’50”E). Laem Son National Park is a marine national park off Thailand’s west coast. The park primarily protects parts of the Andaman Sea, but also contains 15 islands and a small part of the mainland. Burmese long-tailed macaques inhabit only two of these islands, PNY and Thao Island. PNY is a small island with an area of 1.7km^2^ and 5.4km of coastline, situated approximately 750m off the cost of Thailand. Thao Island has an area of 1.3km^2^ and 5.3km of coastline. The islands’ shores consist of sandy beaches, rocky shores, and mangrove, which contain a wide variety of marine invertebrates and shore plants that the macaques process with stone tools and consume daily.

### Identifying Variation

#### Subjects

Our subjects were studied using video that was recorded between 2007–2012, from which we had 90 subjects, consisting of 31 adult females, 17 adult males, 7 adolescent females, 2 adolescent males, and 33 juveniles whose sexes could not always be discerned from video. These subjects were a subset of a population of macaques living on PNY and Thao Island in LSNP. A survey conducted in 2011 reported that there were approximately 192 macaques living in 9 groups on PNY [40]; however the macaques on Thao Island were not well counted, but were approximated to be around 60–100 animals. Based on these numbers, our sample of subjects represents about a third of the studied population.

143 of the macaques on PNY were individually identified and named in 2011 [40], which allowed us to match digital photographs of these named individuals with some of the individuals in our video data. However, as most of the video data was collected three to four years before the identification of individuals, not all the macaques in the videos were present in the population in 2011. However, we were able to discriminate these unnamed individuals on video by their unique facial characteristics, and thus were able to reliably identify all subjects used in this study.

#### Collection of Video

Several researchers, MG, MK, DV, and AT, collected video recordings of tool-using behaviour during several research trips to LSNP in 2007, 2008, and 2012, and obtained 22 hours of video in total across 21 days (~1hr/day). MG, MK, and DV collected video in 2007 between December 1^st^— 5^th^, and in 2008 between February 26^th^—March 2^nd^, June 3^rd^— 23^rd^, and December 10^th^— 20^th^, while AT collected video in 2012 from May 18^th^- 23^rd^. During these trips, researchers circumnavigated PNY and Thao Islands by long-tail boat searching for macaques on the shore. When macaques were located, we stopped the boat and observed them. During some observation periods we recorded instances of tool use with a video recorder, and these videos were used to score our data.

We collected approximately two hours of video using a focal sampling protocol; however, most of our videos were collected without randomized decision rules for sampling. In 2007 and 2008, individuals were not recognizable and so no standard protocols by individual macaque could be designed. However, effort was made to video as many different animals as possible when videoing macaques on the shore. During this phase, there were no standardized stopping procedures for ending a video recording. We tried to video tool-use bouts to completion; however boat and sea conditions could disrupt video sequences by motion, causing the target animal to go off the recording screen for short periods. In 2012, individual macaques could be identified, and AT collected video according to a focal sampling protocol. Randomized lists with the names of all individuals were generated, and individuals were videoed for 5 minutes each time, according to the orders on the lists. If at the end of the 5 minutes the focal subject was in the middle of a tool use bout, we recorded the bout to completion, and the focal was continued until the subject consumed the food item, or ceased to interact with the food item for more than 10s.

#### Video Scoring of Tool-Use Bouts

**Fig 1:**
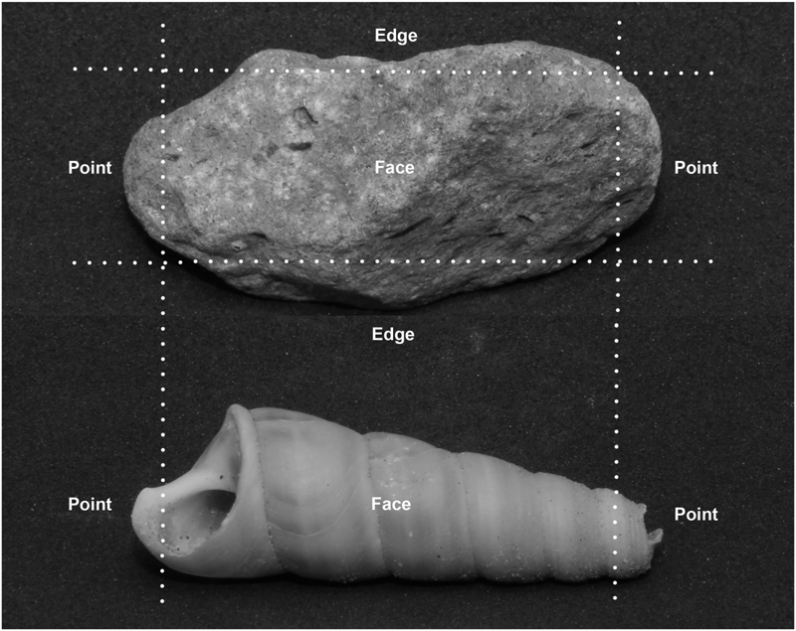
Photographs of stone and shell tools with tool surfaces labeled: P—points, E—Edges, and F—faces. The use of shell tools was scored as either point or face only.

All videos collected were scored by AT, with a standardized procedure that involved defining discrete tool-use bouts, identifying the tool-using individual performing the bout, and scoring the material and behavioural elements of each bout. A bout of tool use commenced when the subject began striking a food item. A bout was scored as ended when the last strike had been applied to the food item before its consumption, or the subject ceasing any interaction with the food item for more than 10s. For each tool-use bout we scored the identity, group membership, age class and sex of the tool user. We also scored the general characteristics of each tool-use bout, which included the duration and number of strikes used. Duration and number of strikes were then used to calculate the strike rate of each bout. We scored several material and behavioural elements for each tool use bout (Table 1). For material elements, we examined the tool and food in each tool-use bout and scored the following, 1) the tool material (i.e., stone or shell), 2) the tool size, which we estimated using a size index based on the size of the tool relative to the macaque’s hand size as they appeared on video, 3) the tool surface used to strike the food (i.e., point, edge, or face; Fig 1), and 4) the category of food (i.e., sessile or unattached). For behavioural elements, we examined the subject’s actions, and scored 1) striking action, 2) strike recoil, 3) tool-hand use, 4) non-tool-hand use, 5) posture, 6) manner of lifting tool, and 7) direction of striking.

**Table 1:** Behavioural and material components of Burmese long-tailed macaque tool use.

Behavioural / Material Component	Levels	Operational Definition
**Striking Action**	*Overhand*	Strikes are executed with the hand(s) brought forward and down.
	*Underhand*	Strikes are executed with the hand(s) brought forward from below the level of the elbow.
	*Clapping*	Strikes are executed by moving the hand horizontally from side to side, to contact the item held in the other hand.
	*Flipping*	Strikes are executed by raising the tool, then flipping it before bringing it down onto the food item.
**Strike Recoil**	*Smooth*	No pause in between strikes and the tool is lifted again immediately after striking the target.
	*Not Smooth*	Pauses between strikes to reposition the tool.
**Hand Use**	*One hand*	Either the left, or right hand is used to handle the tool.
	*Both hands (symmetrical)*	Both hands are used to handle the tool, and both hands carry out the same action.
	*Both hands (asymmetrical)*	Both hands are used to handle the tool, but each hand carries out a different action.
**Non-Tool Hand**	*Support Body*	The non-tool hand is either placed against the substrate to support the body’s weight, or held out to provide balance while striking.
	*Support Food*	The non-tool hand is placed beside the food item to keep it in place, or constantly repositions the food item between strikes.
	*Hold Substrate*	The non-tool hand holds the movable substrate to which the food target is sessile.
	*Hold Food*	The non-tool hand holds the food item, the target is not placed on an substrate.
**Grip Type**	*Power*	The tool is held between the fingers and the palm. The palm is active in squeezing the tool securely.
	*Precision*	The tool is held between the fingers and the thumb. The palm is not active in squeezing the tool, and may only provide passive support.
**Posture**	*Sitting*	The monkey is sitting as it strikes the food item.
	*Standing (Tripedal)*	The monkey stands on three legs as it strikes the food item.
	*Standing (Bipedal)*	The monkey stands on its two hind legs as it strikes the food item.
	*Rising*	The monkey rises from a sitting posture into a bipedal standing posture before striking the food item.
**Tool Lift**	*Fully Lifted*	The tool is lifted entirely off the substrate before being brought down onto the food item.
	*Partially Lifted*	Only one side of the tool is lifted and brought down onto the food item, while the other remains in contact with the substrate.
**Tool Size Index**	*1*	< 1 hand size
	*2*	1–2 hand sizes
	*3*	2–3 hand sizes
	*4*	3–4 hand sizes
	*5*	> 4 hand sizes
**Tool Material**	*Stone*	A stone is used as the tool to process the food item.
	*Shell*	A shell is used as the tool to process the food item.
**Usage Pattern**	*Point*	The edges along the width of the stones, corners, or protruding points of the stones were used to strike the food item.
	*Edge*	The edges along the length of the stones were used to strike the food item.
	*Face*	The broad flat surfaces of the stones spanning the length and width of the stone were used to strike the food item.
**Strike Direction**	*Same Plane*	The tool is directed at a target located on the same plane on which the animal is positioned.
	*Different Plane*	The tool is directed at a target located on a different plane from which the animal is positioned (e.g. vertical face of a large rock, on the underside of an overhanging rock)
**Food Type**	Sessile	Food item is attached to a substrate (e.g. rock, root of a tree)
	Non-Sessile	Food item is not attached to any substrate
**Tool Bout Characteristics**	Bout Duration	Time in seconds, between the first strike made to the food item, and the last strike before the food item was consumed, discarded, or no longer attended to.
	No. of Strikes	The total number of strikes made to the food item before it was consumed, discarded, or no longer attended to.
	Strike Rate	Obtained by dividing the total number of strikes made to the food item, by bout duration.

#### Classification of Tool-Use Bouts

Every tool-use bout was classified into a *hammering class* and *action pattern* (Table 2). Hammering class assignment was determined by identifying which surface of the tool was used to strike the food target. These were, 1) *point hammering*, 2) *edge hammering* and 3) *face hammering*. If a macaque used an auger shell (*Turritella attenuata*) as a tool, we classified only point or face hammering, due to the shell’s conical shape.

**Table 2:** The hammering classes and tool-use action patterns of Burmese long-tailed macaques.

*Hammering Class* / Action Pattern	Description
*Point Hammering*	*Hammering in which the corners and pointed protuberances of a tool are used to strike food items*.
Unimanual Point Hammering	Point hammering where the tool is held in either the left or right hand.
Bimanual Point Hammering	Point hammering using two hands, where both hands handle the tool with the same grip and exert similar force in striking the food item.
Unimanual Supported Point Hammering	Unimanual point hammering where the food item is attached to a mobile rock that is supported by the non-striking hand.
Unimanual Jab Point Hammering	Point hammering where a large tool stone is held on both edge sides with both hands, and an underhand striking action is used.
Unimanual Shell Point Hammering	Unimanual point hammering in which a shell is used as a tool instead of a stone.
*Face Hammering*	*Hammering in which the faces of a tool*, *i*.*e*. *the flat open surfaces of the stone that spanned the full length and width of stones were used to strike food items*.
Unimanual Face Hammering	Face hammering where the tool is held in either the left or right hand.
Bimanual Symmetrical Face Hammering	Face hammering using two hands, where both hands handle the tool with the same grip and exert similar force in striking the food item.
Bimanual Asymmetrical Face Hammering	Face hammering using two hands, where one hand grips the stone and provides downward force during striking, while the other supports the stone and assists in lifting the tool.
Bimanual Stand Face Hammering	Symmetrical bimanual face hammering where the macaque rises from a sitting position, to stand bipedally before striking the food item.
Unimanual Fulcrum Face Hammering	Face hammering where one end of the stone is lifted with one hand, and the other end remains in contact with the substrate.
Bimanual Fulcrum Face Hammering	Face hammering where one end of the stone is lifted with both hands, and the other end remains in contact with the substrate.
Bimanual Asymmetrical Flip Face Hammering	Asymmetrical bimanual face hammering where the stone is raised and flipped before it is brought down onto the food item.
Unimanual Clap Face Hammering	Face hammering where a stone and a food item is held in each hand and clapped together.
Unimanual Shell Face Hammering	Unimanual face hammering in which a shell is used as a tool instead of a stone.
*Edge Hammering*	*Hammering in which the edges of a tool*, *i*.*e*. *the sides along the lengths and widths of the tools were used to strike food items*.
Unimanual Edge Hammering	Edge hammering where the tool is held in either the left or right hand.
Bimanual Symmetrical Edge Hammering	Edge hammering using two hands, where both hands handle the tool with the same grip and exert similar force in striking the food item.
Bimanual Asymmetrical Edge Hammering	Edge hammering using two hands, where one hand grips the stone and provides downward force during striking, while the other supports the stone and assists in lifting the tool.

All tool-use bouts were further sub-divided into an action pattern within the hammering class. Within each hammering class, we first divided action patterns based on one material element, the tool material, and thus were assigned as stone-hammering or shell-hammering action patterns. We then divided action patterns according to hand use, into whether they were unimanual, involving only one hand handling the tool, or bimanual, involving both hands handling the tool. For unimanual action patterns, we then further divided action patterns according to whether the non-tool-handling hand performed a supportive function on the substrate on which food items were attached. For bimanual action patterns, we further divided action patterns into symmetrical patterns, where both hands performed the same action in handling the tool, and asymmetrical, where one hand was dominant in lifting and lowering the tool during striking and the other hand performed a supportive function on the tool. Lastly, we differentiated action patterns according to the dynamics of strike actions used, that are, 1) the manner in which the tool was lifted, whether lifted fully or only partially from one end, 2) the strike action used, whether overhand, underhand, a clapping motion, or a flipping of the stone at the apex of the strike trajectory and 3) posture while striking, whether the macaque remained in a static posture and struck by moving only the arms, or rose to a bipedal posture before each strike to utilize its body to generate strike force. All action patterns were a unique combination of these elements.

#### Analysis of Hammering Class Differences

We collated data on tool-bout characteristics, material, and behavioural elements of each tool use bout by hammering class, and then tested for differences amongst the hammering classes. For tool-bout characteristics, we tested whether there were differences across hammering class for bout duration, number of strikes and strike rate. For material and behavioural elements, we tested whether hammering classes differed on tool size, and whether hammering classes were associated with food type, grip type, hand use, strike recoil, posture, and strike direction. For the latter comparisons on associations between food type, grip type, hand use, strike recoil, posture, and strike direction and hammering classes, we calculated the proportion of tool-use bouts in which there was sessile food type, use of precision grip, use of both hands, smooth recoil, sitting posture, and strike direction towards different plane for each individual across all three hammering classes (See Table 1 for definitions of these element variables), and tested if these proportions differed between hammering classes. Lastly, we also tested for differences within hammering classes, between bouts performed on sessile and unattached foods, for classes where use on both food types was common. Testing these differences were used for comparing with previous descriptions of axe and pound hammering, which were discussed to be largely associated with sessile and unattached foods, respectively.

We used a Kruskal-Wallis H test to test for main effects between hammer class and tool-bout characteristics, material, and behavioural elements, and Mann-Whitney U tests for post-hoc tests, and tests for differences between bouts on sessile and unattached foods in face and edge hammering classes. We chose non-parametric statistical tests because our data did not meet the assumptions required for parametric statistics. We set *p*-value at 0.05, and for post hoc tests we used a Bonferroni adjustment with a calculated significance level of *p* = 0.017 (i.e., 0.05 divided by 3 comparisons).

### Investigating Behavioural and Intergroup Differences

#### Collection of Scan Samples

MG collected scan samples [41] between January 17^th^ and June 24^th^, 2011 from 132 different macaques living on Piak Nam Yai Island. During this time period, MG circumnavigated the island by long-tail boat with a driver on 89 days. When a group of macaques was spotted on the island’s shores, the boat was stopped and anchored to allow for observations. MG scanned each individual present and recorded the individual’s identity and activity at the time into an Olympus DM-5 audio recorder. A macaque was scored as resting, traveling, engaged in social activity, or feeding. If feeding, the macaque was scored for whether they were carrying or using a stone as a tool. If the individual was engaged in tool use, we also recorded the type of food being processed, the part of the tool being used, hand use, posture, direction of striking, and the type of action. The type of action for each tool using scan was scored as axing, pounding, clapping, fulcrum pounding, flip pounding, stand pounding, jabbing, shell pounding or shell picking. We used the variables scored during scan sampling to recode each tool-use scan sample into our hammering class and action pattern classification system.

#### Analysis of Scan Samples

We obtained 3202 scan samples from 132 individuals. However, we excluded 23 individuals with fewer than 10 scan samples, leaving a sample of 3077 scans from 109 individuals for an average of 28 scans per individual, ranging between 10 to 53 scans per individual. Our sample was over half of the ~200 animals from Piak Nam Yai at the time of study [40]. From these scans, we calculated the proportion of individuals ever observed using tools during scan samples. For each tool-using individual, we calculated what proportion of total scans were feeding scans, what proportion of feeding scans were tool-use scans, and what proportion of tool-use scans used each hammering class and action pattern. We also calculated how many types of hammering classes and action patterns each individual used during all scans.

We used these scans to assess how often each hammering class and action pattern was utilized, and we performed comparisons across groups. The 109 individuals observed belonged to groups BC, MN, RF, RS and SS [40]. We first calculated for each individual, the proportion of tool-use scans in which each hammering class was used. Then, we tested if across groups, the proportion of tool-use scans in which individuals’ used each hammering class differed, To do this, we used a Welch’s one-way ANOVA to determine if groups differed in the proportion of tool-use scans in which each hammering class was used, with Games-Howell post-hoc tests to identify which groups differed from each other in each hammering class.

## Results

### Hammering Class and Action Patterns

From the 22 hours of video collected, we investigated tool use bouts and categorized all bouts into hammering classes and action patterns. Our data set included 638 bouts of tool use across 90 individuals, totaling 45 minutes of tool use. This was an average of about 7 tool-use bouts per individual (SD = 8.27), ranging from 1–43 bouts per individual. Tool use bouts ranged between 1s to 60s long, at an average of 4.17s per bout (SD = 5.42). All tool-use bouts were categorized into one of the three hammering classes (Table 2). We observed 398 bouts of point hammering, 147 bouts of face hammering, and 93 bouts of edge hammering. We also identified 17 action patterns (S1 Video). Five of these action patterns were in the point hammering class, nine in the face hammering class, and three in the edge hammering class.

### Differences across Hammering Class

**Fig 2:**
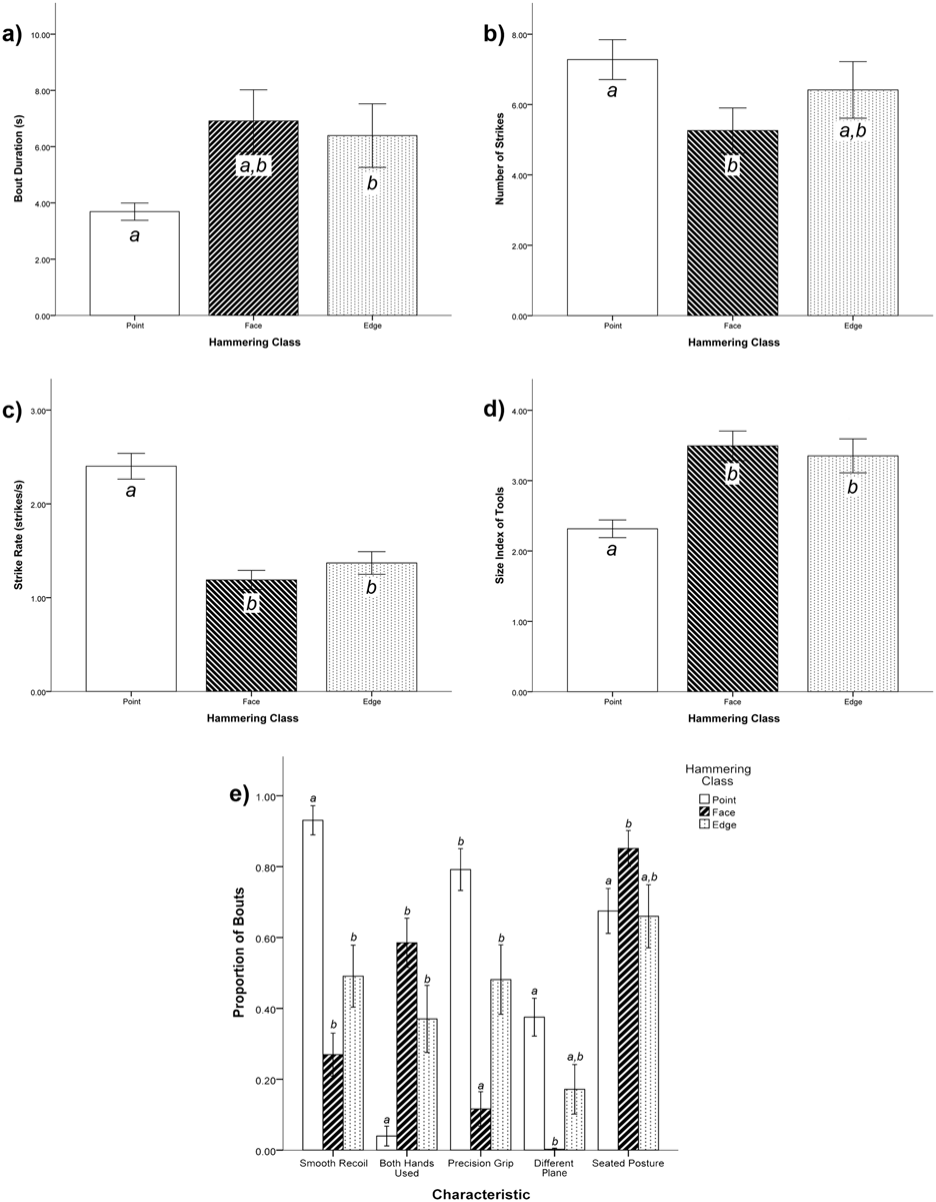
Our data indicated differences in behavioural elements across hammering classes in average a) bout duration, b) number of strikes, c) strike rate, and d) size. Panel e) shows that the proportion of bouts exhibiting smooth recoil, the use of both hands, the use of precision grips, striking towards a different plane, and seated posture differed across hammering classes as well. Letters are used to denote statistical significance. Each letter groups bars into non-significant groups within each analysis, and thus significantly different bars to not share letters. Error bars represent one standard error of the mean.

We found significant differences across the hammering classes for all variables tested. Due to several variables being tested we have organized the results and statistics into tables and figures (Table 3 and Table 4; Fig 2). We found several significant associations. Point hammering was more associated with sessile foods than edge hammering and face hammering, and edge hammering was more associated with sessile foods than face hammering. Tools used for face and edge hammering were larger than tools used for point hammering. Point hammering bouts were shorter than edge hammering bouts. More strikes were used during point hammering than face hammering bouts. Strike rate was faster for point hammering than for edge and face hammering. Point hammering had more smooth recoil of striking than edge and face hammering. Point hammering was less often bimanual than edge and face hammering. Point and edge hammering used precision gripping more than face hammering. In point hammering, striking was directed towards a different plane more than edge and face hammering bout. Face hammering was more often from a seated posture than point and edge hammering.

**Table 3:** Means and standard deviations for all variables for each hammering class.

Hammering Class	N	Sessile Food Type^1^	Tool Size^2^	Bout Duration (s)	Number of Strikes	Strike Rate (Strikes/s)	Smooth Strike Recoil^1^	Both Hands Used^1^	Precision Grip Used^1^	Striking towards Different Plane^1^	Seated Posture while Striking^1^
**Point**	50	0.97 ± 0.23	2.31 ± 0.88	3.69 ± 2.17	7.28 ± 4.01	2.40 ± 0.97	0.93 ± 0.29	0.04 ± 0.20	0.79 ± 0.42	0.38 ± 0.38	0.68 ± 0.45
**Face**	44	0.21 ± 0.34	3.49 ± 1.40	6.91 ± 7.35	5.26 ± 4.26	1.22 ± 0.66	0.27 ± 0.40	0.59 ± 0.46	0.11 ± 0.32	<0.01 ± 0.02	0.85 ± 0.34
**Edge**	27	0.55 ± 0.45	3.35 ± 1.25	6.39 ± 5.88	6.42 ± 4.18	1.37 ± 0.63	0.51 ± 0.45	0.37 ± 0.49	0.49 ± 0.51	0.17 ± 0.38	0.66 ± 0.47

^1^ Numerical values are the average proportions of individuals’ tool-use bouts in which the characteristic in the column header was recorded.

^2^ The average tool size is calculated using a tool size index (Table 1). The average tool size by size index across all styles is 3.05.

**Table 4:** Results of Kruskal-Wallis H and Mann-Whitney U tests for differences between hammering classes.

Variable	Kruskal-Wallis H	Mann-Whitney U		
		*Point vs*. *Face*	*Point vs*. *Edge*	*Face vs*. *Edge*
Food Type	H_2,121_ = 58.47, P < 0.01	U = 150.00, Z = -7.78, P < 0.01	U = 400.50, Z = -3.75, P < 0.01	U = 383.50, Z = -2.74, P < 0.01
Tool Size	H_2,121_ = 21.24, P < 0.01	U = 571.00, Z = -4.10, P < 0.01	U = 347.50, Z = -3.64, P < 0.01	not significant
Bout Duration	H_2,121_ = 7.64, P < 0.02	not significant	U = 442.00, Z = -2.49, P < 0.02	not significant
No. of Strikes	H_2,121_ = 15.19, P < 0.01	U = 598.00, Z = -3.81, P < 0.01	not significant	not significant
Strike Rate	H_2,121_ = 50.11, P <0.01	U = 246.50, Z = -6.39, P < 0.01	U = 180.50, Z = -5.28, P < 0.01	not significant
Strike Recoil	H_2,121_ = 44.84, P < 0.01	U = 295.00, Z = -6.56, P < 0.01	U = 330.00, Z = -4.11, P < 0.01	not significant
Hand Use	H_2,121_ = 36.57, P < 0.01	U = 427.00, Z = -6.16, P < 0.01	U = 452.00, Z = -3.79, P < 0.01	not significant
Grip Type	H_2,121_ = 41.03, P < 0.01	U = 325.00, Z = -6.48, P < 0.01	not significant	U = 382.50, Z = -1.84, P < 0.01
Strike Direction	H_2,121_ = 41.36, P < 0.01	U = 472.50, Z = -2.78, P < 0.01	U = 414.00, Z = -2.98, P < 0.01	not significant
Posture	H_2,121_ = 7.87, P < 0.02	U = 777.50, z = -2.82, P <0.01	not significant	not significant

**Fig 3:**
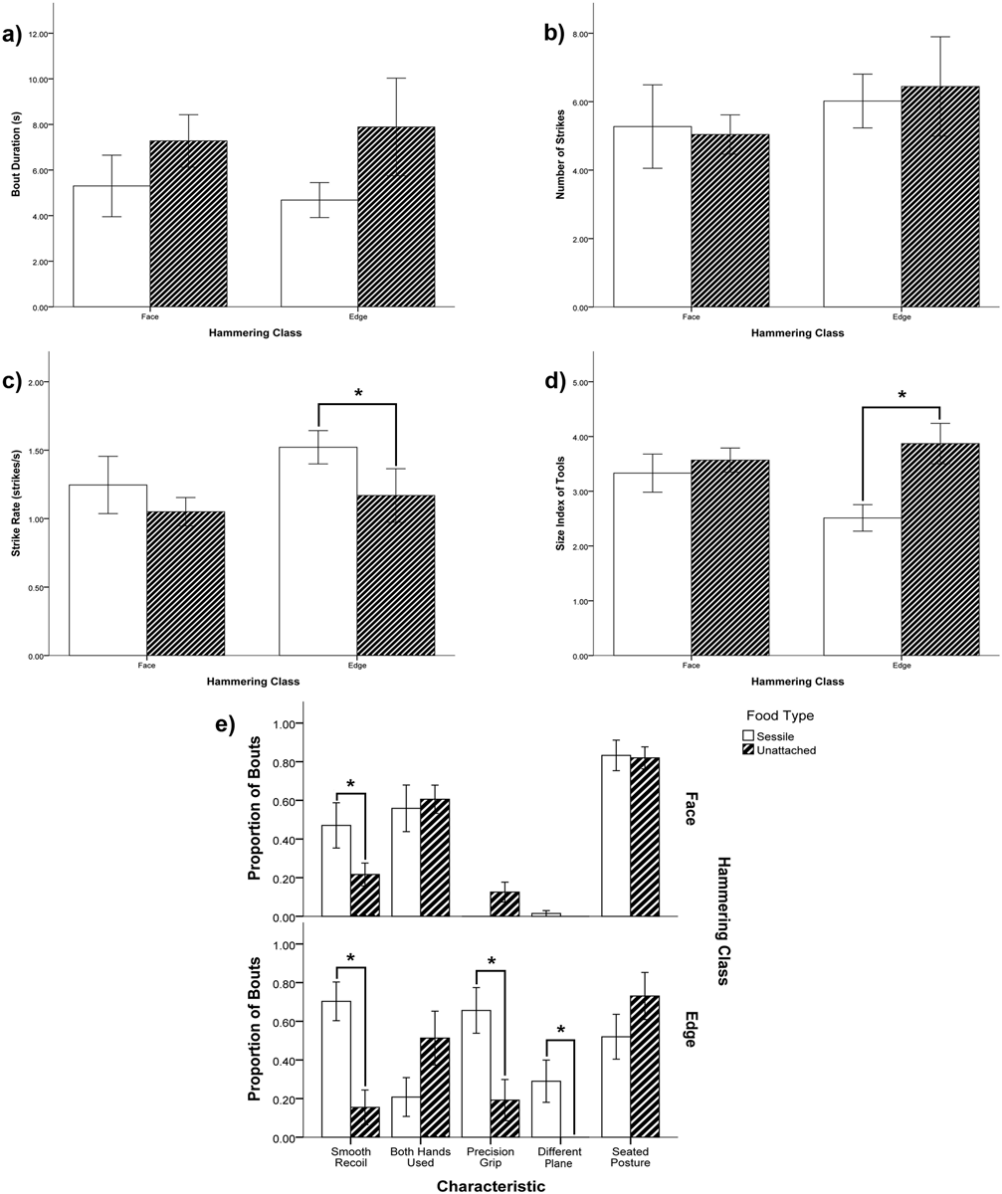
A comparison between use on sessile and unattached foods within both face and edge hammering classes showed differences in behavioural elements across food types within edge hammering, and less so in face hammering. Edge hammering on sessile food resembled axe hammering as c) strike rate was faster, d) tools used were smaller, and e) a higher proportion of bouts exhibited smooth recoil, precision grips, and striking towards a different plane. Face hammering on both food types resembled pound hammering, except in panel e) face hammering on sessile foods had a higher proportion of bouts with smooth recoil than on unattached foods. Asterisks indicate significantly different bars. Error bars represent one standard error of the mean.

We compared tool bouts on sessile and unattached foods within each class. However, point hammering was not assessed because 97% of bouts were on sessile foods, and thus point hammering was almost entirely performed on sessile foods at LNSP. Edge hammering and face hammering did have sufficient cases of sessile and unattached food tool bouts for analysis (Face hammering: 21% sessile, 89% unattached; Edge hammering: 55% sessile, 45% unattached). We found more significant differences of variables determined between hammering on sessile versus unattached foods for edge hammering than for face hammering (Table 5 & Table 6; Fig 3). Edge hammering on sessile oysters had smaller tool sizes, faster strike rates, and a higher proportion of bouts with smooth recoil, precision grip utilization, and striking towards a different plane than edge hammering on unattached foods. Face hammering on sessile foods differed from unattached foods only in having a higher proportion of bouts with smooth strike recoil.

**Table 5:** Means and standard deviations for all variables when face and edge hammering were carried out on sessile and unattached food types.

Hammering Class / Food Type	N	Tool Size^2^	Bout Duration (s)	Number of Strikes	Strike Rate (Strikes/s)	Smooth Strike Recoil^1^	Both Hands Used^1^	Precision Grip Used^1^	Striking towards Different Plane^1^	Seated Posture while Striking^1^
**Face / Sessile**	17	3.33 ± 1.44	5.30 ± 5.57	5.27 ± 5.03	1.32 ± 0.83	0.47 ± 0.48	0.56 ± 0.50	0	0.01 ± 0.06	0.83 ± 0.33
**Face / Unatt.**	41	3.57 ± 1.41	7.28 ± 7.36	5.04 ± 3.68	1.08 ± 0.65	0.22 ± 0.38	0.61 ± 0.47	0.13 ± 0.33	0	0.82 ± 0.36
**Edge / Sessile**	16	2.51 ± 0.97	4.68 ± 3.07	6.02 ± 3.15	1.52 ± 0.49	0.75 ± 0.37	0.21 ± 0.40	0.66 ± 0.67	0.29 ± 0.44	0.52 ± 0.46
**Edge / Unatt.**	13	3.87 ± 1.33	7.89 ± 7.72	6.40 ± 5.24	1.17 ± 0.71	0.15 ± 0.71	0.51 ± 0.50	0.19 ± 0.38	0	0.73 ± 0.43

^1^ Numerical values are the average proportions of individuals’ tool-use bouts in which the characteristic in the column header was recorded.

^2^ The average tool size is calculated using a size index (Table 1). The average tool size by size index across all styles is 3.05.

**Table 6:** Results of Mann-Whitney U tests for differences within face and edge hammering when carried out on sessile and unattached food types.

Variable	Mann-Whitney U	
	*Face (Sessile vs*. *Unattached)*	*Edge (Sessile vs*. *Unattached)*
Tool Size	not significant	U = 40.50, Z = -2.83, P < 0.01
Bout Duration	not significant	not significant
Number of Strikes	not significant	not significant
Strike Rate	not significant	U = 53.00, Z = -2.24, P < 0.03
Strike Recoil	U = 192.50, Z = -2.98, P < 0.01	U = 27.59, Z = -3.45, P < 0.01
Hand Use	not significant	not significant
Grip Type	not significant	U = 52.50, Z = -2.54, P < 0.02
Strike Direction	not significant	U = 65.00, Z = -2.42, P < 0.02
Posture	not significant	not significant

### Prevalence of Hammering Class and Action Patterns

Out of the 109 individuals studied in scan sampling, 87 individuals, or 79.82% of sampled individuals were recorded to use tools (85.55% of adults and adolescents, and 46.67% of juveniles). We found that macaques carried or used tools in 22.25% of their scan samples, which was 48.32% of scans during feeding scan samples. Out of all tool-use scan samples, the proportion of face hammering was 50.08%, point hammering was 41.90%, and edge hammering was 2.41%. Surface use could not be recorded in the remaining 5.61% of tool-use scan samples due to issues of visibility. We found that, in total, 54 individuals point hammered, 68 individuals face hammered, and 12 individuals edge hammered. 49 individuals were observed in their scan samples to use one hammering class, 29 individuals were observed using two, and two individuals were observed using all three hammering classes of tool use.

Tool-use action patterns also differed in the number of individuals observed to perform them, and how prevalent they were in the population (Table 7). Individuals utilized between one and four different action patterns. Of the 87 tool users, 38 individuals utilized only one tool-use pattern, 30 utilized two, 13 utilized three, and six utilized four different action patterns. All action patterns were observed during scan sampling, except supported point hammering and symmetrical and asymmetrical bimanual edge hammering.

**Table 7:** The number of individuals observed to use each hammering class and action patterns from scan samples, the percentage of the population that this constitutes, and average percentage of individuals’ tool-use scans in which each pattern was recorded.

*Hammering Class* / Action Pattern	Individuals	% Population	% Tool-Use Scans
*Point Hammering*	*54*	*60*.*00*	*41*.*90*
Unimanual Point Hammering	52	57.78	37.07
Bimanual Point Hammering	3	3.33	3.11
Supported Point Hammering	Not observed during scan sampling		
Bimanual Jab Point Hammering	1	1.11	1.11
Unimanual Shell Point Hammering	3	3.33	0.53
*Face Hammering*	*68*	*75*.*56*	*50*.*08*
Unimanual Face Hammering	33	36.67	13.28
Bimanual Symmetrical Face Hammering	39	43.33	27.94
Bimanual Asymmetrical Face Hammering	2	2.22	0.40
Bimanual Stand Face Hammering	5	5.56	1.09
Unimanual Fulcrum Face Hammering	4	4.44	3.36
Bimanual Fulcrum Face Hammering	3	3.33	2.19
Bimanual Asymmetrical Flip Face Hammering	2	2.22	1.18
Unimanual Clap Face Hammering	1	1.11	0.37
Unimanual Shell Face Hammering	2	1.22	0.48
*Edge Hammering*	*12*	*13*.*33*	*2*.*41*
Unimanual Edge Hammering	12	13.33	2.41
Bimanual Symmetrical Edge Hammering	Not observed during scan sampling		
Bimanual Asymmetrical Edge Hammering	Not observed during scan sampling		

### Group Differences

**Fig 4:**
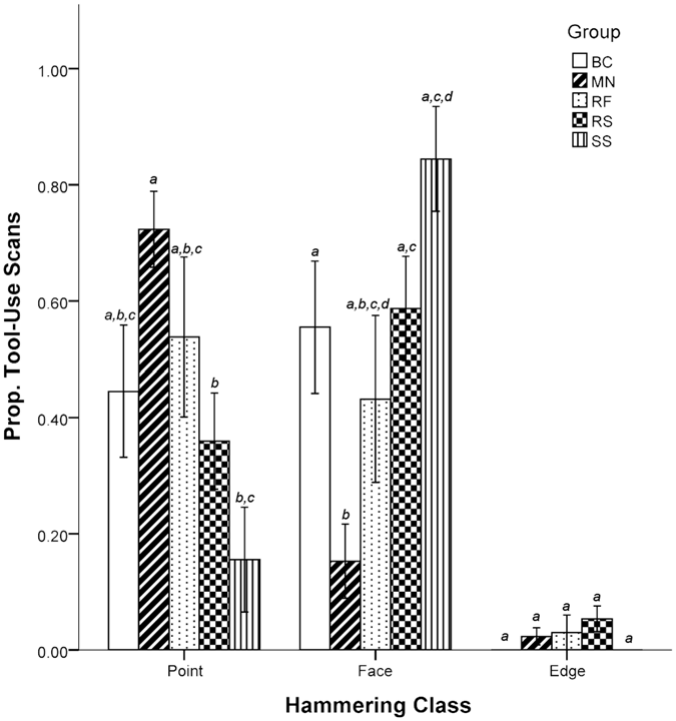
Scan sampling indicated differences in the proportion of point, face, or edge hammering used across five groups. Letters are used to denote statistical significance. Each letter groups bars into non-significant groups within each analysis, and thus significantly different bars do not share letters. Error bars represent one standard error of the mean.

There were differences between groups in the use of hammering classes. The proportions of tool-use scans in which point and face hammering were recorded between some groups were significantly different, but not in edge hammering (Fig 4). Point hammering was used in a significantly higher proportion of tool-use scans in the MN group than in the RS and SS groups. On the other hand, face hammering was used in a significantly higher proportion of tool-use scans in the SS group, and the proportion of face hammering tool-use scans in the MN group was significantly lower than all other groups except the RF group which did not differ significantly from any other group.

## Discussion

Our study was an effort to categorize macaque tool use based on its material and behavioural elements. In particular, we wanted to develop a systematic ethogram of macaque tool-use action patterns that could facilitate future study. We categorized macaque stone-tool use by hammering class, which was based on the surface of the tool that was used to strike food items, these being point hammering, face hammering, and edge hammering. Furthermore, we were able to discretely identify 17 action patterns within these hammering classes based on different patterns of elements. The common action patterns were *unimanual point hammering* (58%), *symmetrical bimanual face hammering* (43%), and *unimanual face hammering* (37%). *Edge hammering* (13%) was less common than point and face hammering. However, *unimanual edge hammering* was not as rare as other action patterns, which were all used by 5% or less of the population. In addition, one action pattern, bimanual jab point hammering, has only ever been observed in one individual.

Action patterns were distinguishable by the unique combinations of material and behavioural elements that comprised them. The only material element that was used to discriminate action patterns was whether a stone or shell was used as a tool, as the other material elements, size of stone and food type could potentially vary in all of the action patterns. Furthermore, we wanted to leave open the ability to test how behavioural actions relate to these material elements, as we acquire more data in the future. As a result, our classification system is primarily a differentiation across behavioural elements, hence why we refer to them as action patterns.

### A Re-Assessment of Axe and Pound Hammering

Previous research had described two basic forms of stone pounding in Burmese long-tailed macaques [12]. These were, “axe hammering”, which was predominantly hammering to crack open sessile rock oysters, and “pound hammering”, which was predominantly hammering to crack open unattached food items placed on anvils [12]. Furthermore, axe hammering was described as a form of hammering most often using the tool’s points, having a faster strike rate, using a precision grip, having a wider range of motion, having smooth strike recoil, and using a variety of postures. In contrast, pound hammering was described as a form of hammering most often using the face, always starting from a seated posture, having a slower strike rate, using a power grip, not usually having smooth strike recoil, and always being performed toward the horizontal plane the animal is seated on and from a seated posture [12]. In this current study, we differentiated patterns of tools in greater detail, and tried to assess the utility of the simple dichotomous discrimination of axe and pound hammering. To do this, we performed an analysis across hammering classes to test how each class related to food type, tool size, bout duration, number of strikes in a bout, strike rate, strike recoil, hand use, grip type, direction of striking, and posture. We then compared our findings to the descriptions of axe and pound hammering used formerly.

Our current analysis of hammering classes was mostly consistent with prior descriptions. Axe and pound hammering most clearly related to our classes of point and face pounding respectively. Furthermore, we found that face and point hammering significantly differed in all elements, except bout duration. In addition, they varied in the ways described previously. That is, point hammering was almost always used for sessile foods, and showed the features that had been described for axe hammering, while face hammering was mostly used for unattached food, and showed features described in pound hammering. More problematic though was the edge hammering class, because edge hammering was regularly observed for both processing sessile and unattached food, and thus edge hammering could be used interchangeably as both axe and pound hammering.

To account for this, we compared edge hammering of sessile foods with that on unattached foods. We performed a similar analysis on face hammering as well, since face hammering also had cases on both sessile and unattached foods. We found that edge hammering on sessile foods strongly resembled axe hammering in its features, while edge hammering on unattached foods resembled pound hammering. In contrast, we did not find that face hammering of sessile foods resembled axe hammering, other than that smooth recoil was significantly more frequent. This shows that food type alone is not driving the axe hammering-pound hammering distinction. Rather sessile foods produce the axing form when the edges or points are used, while face hammering always resembles the pounding form of tool use, regardless of food type. Previous research discussed difference in the “precision” of use of axe and pound hammering, arguing that axe hammering was a more precise form of tool use compared to pound hammering [12]. Hammering of any food item with the face, or an unattached food source with the edge is a less precise form of hammering, because it does not require a specific point of the tool to strike a specific area on a rock. There is also less specificity of where on the edge or face the stone must hit the food item as these surface areas are larger, and furthermore, the macaques can manipulate an unattached food item to a convenient location on an anvil. Axe hammering differs because when chipping oysters with the edge or point of a tool, a small region of the stone must strike at a non-manipulable target attached to a rock. Substrates on which sessile oysters are attached to can be oriented at many different angles, for example, an overhanging rock, requiring the axe hammering macaque to alter its strike angle according while maintaining accuracy. From such action, a more precise form of tool use results, called axe hammering, which has not been documented in any other non-human primate.

Across hammering classes we found significant differences in the size of tool used. Size is a material element that has been discussed to differ between axe and pound hammering, and indeed tool size does relate to sessile and unattached foods. Our results showed that macaques, on average, used larger tools for face and edge hammering than point hammering. The averages do not reflect all use though, and smaller tools were regularly used for unimanual action patterns of face and edge hammering, and thus what we can conclude is that during unimanual tool use, macaques are using small hand-sized tools regardless of the surface being used. However, face and edge hammering more often involved the use of both hands for tool handling compared to point hammering, and thus stones were on average larger in the face and edge hammering classes. Exceptional cases were bimanual point pounding and bimanual jab hammering action pattern, which were always carried out with large or extra large tools held with both hands. This further supports that tool size and hand use were strongly related during our study. In addition, this shows that when discriminating axe hammering and pound hammering the size of the stone was not the critical component, as the bimanual forms of point hammering with a large stone resembled all other features of axe hammering, while unimanual pounding with small stones still resembled the features of pound hammering.

Gripping significantly differed across the hammering classes. Macaques used power grips to handle tools in a significantly larger proportion of face hammering, than both edge and point hammering. Precision gripping was largely associated with sessile foods, as when edge hammering broken into sessile and unattached components, precision grips were used significantly more often with sessile foods than with unattached. Moreover, point hammering, which is primary performed on sessile foods, was predominantly performed with a precision grip. An exception to this was shell point hammering with auger shells (*Turritella attenuata*), which are handled with a vertical squeeze power grip. This grip relates to the conical shape of the tool, and is performed in a motion and grip similar to overhand stabbing. In this study, all shell tools observed were auger shells. However, other shells such as dog and crown conches (*Laevistrombus canarium* and *Pugilina cochlidium*) or tun shells (*Tonnidae*—probably *Tonna sp*.) have also been observed used as tools and are shaped much differently and more like a stone. We do not have any data on the usage patterns of these much more rare types of shell tools.

Some of the features of striking significantly varied across the classes, and also were mostly comparative with the previous axe and pound-hammering dichotomy. Strike rates were significantly faster during point hammering than both face and edge hammering. The recoil of the arm following a strike was significantly more often smoothly returned to starting point, rather than involving fumbling or repositioning of the tool, during point hammering than face and edge hammering. Furthermore, during point hammering, striking was significantly more often directed towards targets at differing spatial orientations from the plane on which the animal was positioned, unlike face hammering strikes, which were almost exclusively targeted horizontally below the user. Lastly, macaques carried out tool use from a seated posture significantly more often during face hammering, than point hammering. During point hammering, we regularly observed macaques standing when point hammering, in order to reach where the sessile target was located. For face hammering, we observed macaques standing bipedally to perform stand face hammering. However, the macaque would always rise from an initially seated posture before standing bipedally, and then return to a seated position following the strike.

### Variation across Groups

In our study we found that each group varied in the frequency of different hammering classes observed. It is this type of variation we seek to explore by using a behavioural catalog of classes and action patterns, and thus we provide preliminary evidence that defining variation of macaque tool use in this way could lead to understanding spatial and temporal changes in its usage. Across the groups of PNY, we found some evidence that habitat may have affected the distribution of differing hammering classes, which was due to the availability of prey in each habitat. Groups that ranged predominantly within mangrove habitats used a higher proportion of point pounding than face pounding action patterns in their scan samples. The reverse was true for individuals in groups whose coastal ranges consisted mainly of rocky shore habitats. Oysters made up a larger proportion of foods processed in mangrove habitats, while some common unattached food types like nerite and tooth-lipped snails were more common on rocky shore habitats than in the mangroves [34]. Since point pounding is strongly associated with sessile foods, it is congruent with habitat to see more of that class of hammering in mangrove habitats, and likewise, more face hammering on the rocky shore. This suggests an influence of ecology on behavioural patterns of stone tool use in Burmese long-tailed macaques.

### Examples of Using a Catalog of Variation

Detailed investigations into the behavioural action patterns within single types of animal tool use are not abundant in the literature. This could be due to an actual lack of sufficient variation in action patterns, or that the variation is there but researchers have focused on only a few variants while ignoring others. There have been broader studies on variation in the presence and absence of different types of behaviours across populations. These studies have allowed the discovery of behavioural traditions by identifying the prevalence of different behaviours across populations that are not clearly the result of ecological differences, and are therefore more likely transmitted via social learning processes [42]. Well-known examples are the documentation of inter-population differences across chimpanzee communities [27] and orangutan populations [43, 44], where a variety of cultural behaviours, including tool use and non-foraging-related behaviours, have been identified based on their presence in some communities and absence in others. In capuchins, it has also been found that groups of *S*. *libidinosus* inhabiting Serra da Capivara have more complex tool kits than groups of capuchins in other locations such as Boa Vista and Tietê Ecological Park [45]. These studies could benefit further from more detailed investigations into more specific aspects of behavioural variation within each type of tool use or behaviour, to better understand how various ecological and social factors shape traditional behaviours in different populations. Furthermore, in species that may not exhibit such a wide variety of different behaviours, this approach to identifying traditions through behavioural differences can only work if behavioural variants within a single type of behaviour are well documented, and this is where a catalog such as ours will come in very useful.

In our study, we have tried to develop a comprehensive list of variation in actions that macaques use during stone tool use, which in many ways is complementary to studies on the behavioural patterns of stone handling in Japanese macaques [46]. The cataloging of variation in action patterns of Japanese macaque stone handling [29] was a foundational step in their work, and provided the basis for an “inter-populational comparative approach”, developmental studies, ecological influences on behaviour, and individual differences [46]. Using their catalog, researchers studied variation from their catalog in 10 populations around Japan, and found that behavioural patterns, or stone-handling action patterns, were patchy in their distribution suggesting local stone-handling traditions [29]. There was greater similarity in geographically closer populations of Japanese macaques, and this was concluded to show cultural zones of these local stone handing traditions. They also were able to look at developmental patterns [33], and found that offspring do not necessarily perform the same behavioural patterns that their mothers do. They argue this was because adult forms of handling were more complex, and that individual learning process played a large role in forming an individual’s stone-handling repertoire.

Currently, we are investigating the distribution of macaque tool use across the Andaman Sea coast in Thailand and Myanmar, and the Thai Gulf coast (Gumert et al., in prep). As we uncover more islands and areas where long-tailed macaques exhibit stone tool use, we will be able to conduct similar cross-population comparisons as have been done in the stone-handling Japanese macaques, and cultural traditions in chimpanzees, orangutans, and capuchins. Our categorization system developed in this study provides the basis for making refined discriminations across populations to detect subtle differences in local behavioural traditions, and identifying such variation in the structure of action patterns across populations will allow us to test how ecological and cultural factors contribute to such variation. We will be able to use the catalog developed in this paper to determine which action patterns are present in each new population discovered, whether there are differences in how each action pattern is in each population, and whether there are new action patterns in the stone-tool-use repertoire of other populations that have not been observed in LSNP.

We have also begun study on the development of stone-tool-use skills a population of tool-using macaques on Koram Island, which is off the Thai Gulf coast in Khao Sam Roi Yot National Park, Prachuap Khiri Khan Province, Thailand (Tan et al. in prep). We have been using this catalog of tool-use action patterns to score tool-use bouts in a study to better understand the effects of social networks on development. Using this technique we will be able to assess whether young macaques develop the same action patterns that are used most commonly by their mothers and other skilled individuals with whom they are most closely affiliated. We will also be able to investigate if closely affiliated individuals share the same action patterns for processing the same food items. This is similar to a study done on capuchins, which showed that individuals that spent more time in proximity to each other were more likely to share similar food processing techniques than less closely affiliated group members [47]. This has also been done with Japanese macaques, where researchers found the young macaques did not acquire the same stone handling patterns as their mothers [33]. By documenting the acquisition of tool use and its various action patterns we will be able to better assess the influences of environment, social learning, and individual trial-and-error learning processes on tool-use development.

### Comparison with Japanese Macaque Stone-Handling and Other Stone-Tool-Users

It will be of high interest to look for homology in the action patterns of stone-handling macaques and stone tool-using macaques, and what these might suggest about how stone-tool use came about in the macaque lineage. For example, the *Pound on Surface* [29] is essentially *Pound Hammering*, without pounding any food, but sometimes pounding other stones. The stone-handling pattern *Clack* [29] is similar to our *Clap Face Pounding* tool-use action pattern, again, except it is two stones that are hit rather than a stone and a food item. Japanese macaques also *Flip* stones [29]. A flipping action is an element of *Flip Face Hammering* in our list of action patterns. The extensive work on stone handling in Japanese macaques, and lesser work on other stone-handling species such as common long-tailed macaques (*M*. *f*. *fascicularis*) [48], Taiwanese macaques (*M*. *cyclopis*) [49] and rhesus macaques (*M*. *mulatta)* [49, 50] suggest basic manipulation of stones is a general propensity in the *Fascicularis*-species group of macaques [48–50], and perhaps this manipulative ability is the basis for Burmese long-tailed macaques becoming tool-users. However, macaque stone-handling is non-functional and has been only observed in provisioned macaques [51], whereas stone tool use is functional and is the only stone-using behaviour ever reported in wild, unprovisioned macaque monkeys. This suggests some very unique circumstances are required to elicit stone-use in free-ranging and wild macaques.

We can question what the basis is for macaques beginning to include stone use into their behavioural repertoires under these conditions, which probably represent novel conditions—e.g., a shift to human environment for stone handling, and a shift to coastal and small island habitats with rocky coasts for stone-tool using. It has been suggested that stone handling may be a response to un-satiated food manipulation because feeding is disrupted by the rapid depletion of the provisioned food [51]. Essentially, food is consumed much faster than it would be under wild conditions, and thus hunger is satiated more quickly leaving more time for non-functional explorative play behaviour during feeding time.

On the other hand, the Burmese long-tailed macaques are foraging on shores and mangroves, where there are numerous rocks and encased foods, and no provisioning. How they started including stones into their feeding behaviour is unknown, but if we take what is known about stone handling behaviour we could surmise that stone use may have emerged through a similar behavioural mechanism. When a macaque tries to process an encased food item without tools, it can often times be a long, and frustrating process, that involves frantically manipulating the object and things around it. We can consider this a disruption to their usual feeding activity caused by an inability to easily open and consume encased food items. This disruption channels the macaque into a greater range of manipulative and explorative behaviours directed towards the food and nearby items. Gumert (personal observation) has observed long-tailed macaques trying to open encased food, both shellfish and coconuts, without tools, and sometimes these episodes can last tens of minutes with many manipulative actions being performed on the item. These episodes often contain frantic manipulative behaviours expressed towards the encased foods, which can be extended to surrounding items, such as leaves, sticks, and stones (Tan, personal observation). These combinatory manipulations may have then led to the opening of foods with stones. However, these are ad lib observations and further study needs to be performed on these rare events to potentially understand how a macaque can develop from opening encased foods by manipulation and direct percussion, to opening them with stone tools.

Comparing the action patterns of long-tailed macaque stone-tool use to similar behaviour in chimpanzees and capuchins is less straightforward because there are no extensive catalogs of behavioural variation in the pounding patterns of these species. This creates the immediate assumption, and potentially an illusion, that chimpanzee and capuchin stone tool use is very homogenous, and lacks significant behavioural variation in terms of action patterns. General descriptions of tool use in chimpanzees report that they crack nuts placed on anvils from a seated posture, striking with hand-sized stone or wooden hammer usually by swinging one arm, or less frequently, with both hands [52]. On the other hand, the prototypical description for capuchins cracking nuts with stones is that the stones are much larger relative to their body size than are chimpanzees, and they strike by rising from a seated to a bipedal posture, lifting the stone with both hands to shoulder level or higher before smashing it onto the nut [25]. Macaques perform action patterns similar to both of these forms of tool use, and we would call the chimpanzee description unimanual and bimanual face hammering, and the capuchin description bimanual stand face hammering.

Researchers of chimpanzee and capuchin tool use have reported that detailed documentation on the variation of stone hammering action patterns has not been carried out (Biro, Fragaszy and Visalberghi, personal communications). However, in capuchins at least, researchers express that individuals may vary in their “style” of tool use, such as jumping forward, and lifting tools to different heights, which may indicate different action patterns that have not been formally documented (Fragaszy and Visalberghi, personal communications). One study on capuchins illustrates that the prototypical stand pounding of capuchins is certainly not the only form of stone tool use they employ, and indeed may have a range of stone actions that seem related to the food and size of stone [22]. Regardless, until more detailed reports appear about the behavioural patterns of chimpanzee and capuchin stone-use, any complete comparisons with macaques on behavioural variation would be premature. However, if we do take the freedom to make a cursory comparison, with the limited information available, we would suggest that macaques, capuchin, and chimpanzees all perform some forms of pound hammering, while only macaques perform axe hammering. The simplest explanation for this difference is the sessile oysters that macaques are processing. However, we must note one anecdotal report on capuchins hammering oyster shells with other oyster shells [53], but descriptions of the behaviour are not clear enough to make any comparisons to our study on hammering classes and action patterns. Moreover, no reports of stone tools to process oysters have been put forth. Another factor to consider is the precision gripping capability of macaques, compared to capuchins and chimpanzees [54]. We eagerly await more detailed descriptions on the behavioural catalogs of chimpanzee and capuchins stone use, which would allow more robust comparison across species.

### Relevance to Primate Archaeology

Work has been done on how different types of macaque stone-tool usage leave different use-wear patterns on macaque tools. Such scarring, or use-wear, on stones has been used to discriminate axe and pounding hammering [12], and has been tested to be a reliable marker of past behavioural use [39]. Investigation of the stones relates to the emerging field of primate archaeology, which studies the evolution of lithic technology by comparing stone use across primates, looking into the histories of non-human stone tools, and comparing human and non-human primate lithics [19, 13, 55–59]. The ethogram of classes and action patterns we have generated here might be able to further contribute to researchers studying the features of use-wear in macaque stone tools. We suspect that different actions might damage stones in different ways. Indeed, the classes are easy to discriminate because use-wear appears on the surface being struck, and has already been used as a basis for categorizing use-wear patterns [12, 39]. Expanding on this would be to study if the different action patterns also produce unique wear patterns that could then allow us to reconstruct in greater detail the behavioural histories of stones used as tools that have been recovered from macaque sites.

## Conclusion

We have systematically identified categories of variation in macaque stone-tool-use at LSNP. At this site, macaque tool use consisted of three hammering classes based on whether the tool’s face, edge, or point was used, and 17 action patterns based on tool material, hand use, postural differences, and striking motions. We performed analyses on hammering class across several material and behavioural elements, which showed distinguishable differences across the classes. These findings supported earlier descriptions of axe and pound hammering, and we confirmed that point hammering and face hammering patterns fit earlier descriptions of axe and pound hammering respectively. However, we found that all edge hammering action patterns were interchangeable between axe and pound hammering, depending on whether the food source was sessile or unattached. We found differences between groups in the prevalence of hammering classes, which showed that inter-group variation is detectable using our catalog and could be used for comparative investigations. Our work relates to a similar catalog of actions patterns developed to describe stone handling patterns in Japanese macaques, however, such detailed catalogs of behavioural action have not yet been reported for other stone-using primates. Developing catalogs of behavioural action for stone-use behaviour, such as ours, will help contribute to future research comparing populations, documenting development, and investigating use-wear patterns.

## Supporting Information

S1 Video Action Patterns in Burmese Long-Tailed Macaque Stone-Tool Use. (M4V)
